# Paralytic ileus in a patient on clozapine therapy showing an inverted clozapine/norclozapine ratio after switching valproic acid to carbamazepine: a case report

**DOI:** 10.1177/20451253241255487

**Published:** 2024-05-31

**Authors:** Geke van Weringh, Leonieke van Koolwijk, Lieuwe de Haan, Daan J. Touw, Mariken B. de Koning

**Affiliations:** Groningen Research Institute of Pharmacy, University of Groningen, Antonius Deusinglaan 1, Groningen 9713 AV, The Netherlands; Department of Clinical Pharmacy, OLVG Hospital, Amsterdam, The Netherlands; Arkin Institute for Mental Healthcare, Amsterdam, The Netherlands; Arkin Institute for Mental Healthcare, Research Department, Amsterdam, The Netherlands; Department of Psychiatry, Amsterdam University Medical Center, Amsterdam, The Netherlands; Groningen Research Institute of Pharmacy, University of Groningen, Groningen, The Netherlands; Department of Clinical Pharmacy and Pharmacology, University Medical Center Groningen, University of Groningen, Groningen, The Netherlands; Arkin Institute for Mental Healthcare, Research Department, Amsterdam, The Netherlands; Department of Psychiatry, Amsterdam University Medical Center, Amsterdam, The Netherlands

**Keywords:** carbamazepine, case report, clozapine, clozapine/norclozapine ratio, fluvoxamine, paralytic ileus, pharmacokinetic drug interaction, valproic acid

## Abstract

This case report examines the possible correlation between the clozapine/norclozapine ratio and the occurrence of constipation and paralytic ileus. We present the case of a 42-year-old patient diagnosed with schizoaffective disorder undergoing clozapine therapy. Despite intensive treatment with clozapine, haloperidol, valproic acid and biweekly electroconvulsive therapy sessions for over a year, florid psychotic symptoms and fluctuating mood swings persisted. Therefore, valproic acid was replaced by carbamazepine, a potent inducer of several CYP450-enzymes. To maintain clozapine plasma levels, fluvoxamine, a CYP1A2-inhibitor, was introduced at a dose of 25 mg before this switch. After addition of carbamazepine, there was a significant decline in clozapine levels, necessitating an increase in fluvoxamine dosage to 50 mg. Five weeks later the patient was admitted to a general hospital with a diagnosis of paralytic ileus. Treatment with enemas proved effective. Drug concentration analysis revealed a 2.5-fold increase in norclozapine levels in the weeks preceding hospital admission, resulting in an inverted clozapine/norclozapine ratio. Treatment with clozapine, carbamazepine and fluvoxamine was continued as the patient demonstrated clinical improvement on carbamazepine. Concurrently, an intensive laxative regimen was initiated. Two weeks later, the patient was readmitted to the general hospital due to suspected paralytic ileus and faecal vomiting, once again displaying an inverted clozapine/norclozapine ratio. We discuss potential mechanisms contributing to the occurrence of the paralytic ileus in this patient, including the antagonism of muscarinic M3 receptors by both clozapine and norclozapine, as well as the agonism of delta-opioid receptors by norclozapine. This case highlights the potential significance of both the clozapine/norclozapine ratio and absolute norclozapine levels as risk factors for constipation and paralytic ileus in patients on clozapine therapy.

## Introduction: Clozapine and constipation/paralytic ileus

Clozapine is the most efficacious antipsychotic drug in treatment-resistant schizophrenia.^1^

Besides this superior efficacy it has a very complex adverse reaction profile. Clozapine has several potentially life-threatening side effects, such as agranulocytosis, diabetic ketoacidosis, constipation and myocarditis. Other side effects include weight gain, metabolic effects, sedation, and hypersalivation.

Constipation is often neglected and underestimated. A meta-analysis of 32 studies established a prevalence of clozapine-associated constipation of 31.2%.^2^ If left untreated, constipation can cause serious complications such as bowel obstruction, paralytic ileus, colon perforation, aspiration of faecal vomitus and bacterial sepsis.^3^

Complications of constipation are among the major causes of death in clozapine patients.^4^ Data from VigiBase, the World Health Organisation’s global database, showed that with 326 deaths out of 2814 cases of ‘broad constipation’ (constipation, toxic megacolon and paralytic ileus) the relative mortality was 12%. Inpatient setting^2^ and anticholinergic co-medication^5^ are co-occurring risk factors for constipation.

Here, we present (following the CARE Guidelines^6^) a patient on clozapine therapy who developed paralytic ileus after switching from valproic acid to carbamazepine. We describe how an altered clozapine metabolism resulting from a pharmacokinetic interaction could have contributed to the occurrence of paralytic ileus.

## Case description

A 42-year-old male smoker of mixed African/Caucasian descent diagnosed with schizoaffective disorder (295.70A, DSM-V) had several admissions to a psychiatric hospital over the last 20 years. Since 2019, he stayed in a specialized long-term closed ward for patients with serious mental illness and extremely disruptive/aggressive or otherwise dangerous behaviour for whom treatment in a regular ward did not result in improvement. The schizoaffective disorder manifested as chronically present florid psychotic symptoms (auditory hallucinations, paranoid and grandiose delusions) and fluctuating mood states, often featuring a manic presentation characterized by an agitated mood. Profound impulse control disturbances were evident, marked by multiple incidents of aggression. Driven by psychotic convictions, the patient severely assaulted his mother.

Clozapine therapy was started in 2005 but several times discontinued, the last time in 2018, because the patient developed severe constipation, leading to hospitalization with suspected ileus. In January 2019, clozapine was restarted and titrated to therapeutic plasma levels. In 2019, plasma levels of clozapine fluctuated between 347 and 672 µg/l, and plasma levels of norclozapine were between 230 and 520 µg/l. Comedication at that time consisted of lithium carbonate (1200 mg at bedtime) and zuclopenthixol long-acting injection (LAI, 200 mg every 2 weeks). In 2020, higher clozapine plasma levels – even above 1000 µg/l – were reached, but severe psychotic symptoms persisted. Zuclopenthixol LAI was substituted with haloperidol LAI (225 mg every 2 weeks, later switched to oral haloperidol), yielding some reduction in dysphoria. Nevertheless, the patient’s psychotic state persisted unaltered. In September 2020, biweekly electroconvulsive therapy (ECT) was initiated. By May 2021, the lithium was stopped due to cognitive side effects, and in September 2021, valproic acid (2 g once daily) was introduced. In 2021, before valproic acid addition, the mean clozapine/norclozapine ratio was 1.99 (mean clozapine plasma levels 957 µg/l and mean norclozapine plasma levels 481 µg/l, *n* = 39). After switching to valproic acid, the mean clozapine/norclozapine ratio increased to 3.15 (mean clozapine plasma levels 716 µg/l and mean norclozapine plasma levels 227 µg/l, *n* = 62). After >1 year of biweekly sessions of ECT in combination with clozapine (plasma levels between 400 and 1300 µg/l), haloperidol (plasma levels between 10 and 15 µg/l) and valproic acid (plasma levels fluctuating around 80 mg/l), only minimal improvement was observed. There had been no further incidents of aggression; however, florid psychotic symptoms and fluctuating mood states persisted. The other drugs used were glycopyrronium 2 mg at bedtime, lactulose 30 ml and memantine 20 mg. Lorazepam and droperidol were used as needed. The patient is an intermediate CYP2D6 metabolizer (CYP2D6 *2/*3) and an extensive metabolizer of CYP1A2 (CYP1A2 *1/*1F), CYP2C9 (CYP2C9*1/*1), CYP2C19 (CYP2C19*1/*1) and CYP3A4 (CYP3A4*1/*1). Patient’s BMI is 22.5 (height 171 cm, weight 65.9 kg).

**Figure 1.**
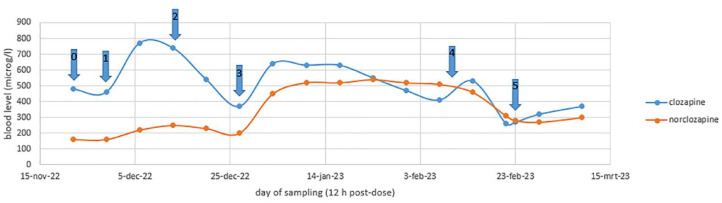
Clozapine and norclozapine levels in time. Arrow 0: ‘baseline’ plasma levels on clozapine 600 mg once daily + valproic acid; arrow 1: addition of fluvoxamine 25 mg, clozapine dose adjusted to 500 mg once daily; arrow 2: valproic acid replaced by carbamazepine; arrow 3: clozapine dose increased to 550 mg once daily, fluvoxamine dose increased to 50 mg; arrow 4: admission to hospital, diagnosed with paralytic ileus; after discharge clozapine dose decreased to 450 mg once daily and carbamazepine dose increased; arrow 5: readmission to hospital with suspected paralytic ileus.

Because of the serious treatment-resistant symptoms, valproic acid was replaced by carbamazepine. As carbamazepine is a strong inducer of several CYP450 enzymes and can decrease clozapine levels, precautionary measures had to be taken. Firstly, fluvoxamine 25 mg was added to the medication to inhibit clozapine metabolism. Clozapine dose was adjusted from 600 to 500 mg once daily at the same time (Figure 1, arrow 1). Two weeks after starting fluvoxamine, carbamazepine (200 mg twice daily) was added and valproic acid was stopped (Figure 1, arrow 2). Clozapine levels decreased the next 2 weeks to 370 µg/l, which was below baseline (460 µg/l before adding fluvoxamine). Therefore, both fluvoxamine and clozapine doses were increased (to 50 mg and 550 mg once daily, respectively) (Figure 1, arrow 3). Patient took his pills under direct supervision of a nurse and always remained in sight for 30 min to avoid non-adherence.

A total of 5 weeks later, the patient was admitted to a general hospital after almost 1 week of no defecation, nausea and vomiting. On the day of admission, eight sachets of macrogol were given, but he immediately vomited. Patient was diagnosed with paralytic ileus caused by clozapine. All oral medication was stopped, metoclopramide (against the nausea) and haloperidol were given intravenously.

Drug concentration levels showed increasing norclozapine in the weeks before hospital admission, and an inverted clozapine/norclozapine ratio 2 days before admission was observed (Figure 1, arrow 4). Clozapine level was 410 µg/l and norclozapine level 510 µg/l.

Carbamazepine plasma levels fluctuated between 5.2 and 8.0 mg/l in the weeks before admission.

Treatment of the ileus was conservative with enemas and showed a good response. Clozapine was restarted the evening after admission, and the next day, the patient was dismissed from the hospital back to the psychiatric hospital. Clozapine dose was reduced to 450 mg once daily to lower the anticholinergic burden. Carbamazepine was continued, and the dose was increased to 600 mg daily because psychiatric symptoms, especially the mood, had improved on carbamazepine. Although the psychosis remained unabated, the patient could, for the first time in years, join a family weekend because he was very friendly and cooperative. An intensive laxative treatment (with macrogol, lactulose, bisacodyl and enemas as needed) was started to prevent constipation.

Two weeks later, the patient was readmitted to the general hospital, suspected of paralytic ileus and faecal vomiting. Again, there was an inverted ratio, with clozapine level being 270 µg/l and norclozapine level 280 µg/l (Figure 1, arrow 5). After admission, the patient had spontaneous defaecation and was dismissed the next day. Glycopyrronium was stopped in the hospital due to its contraindication paralytic ileus. No hypersalivation was observed after stopping the glycopyrronium tablets.

## Discussion

**Figure 2.**
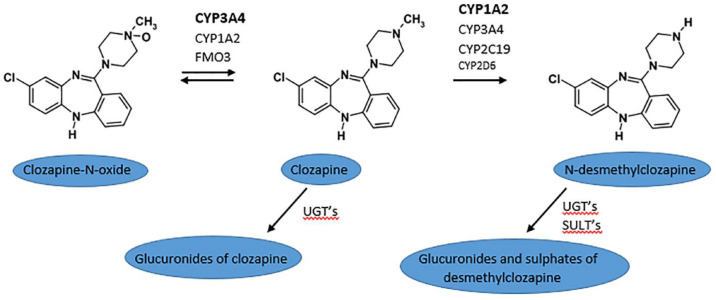
Clozapine metabolism. In bold, the main enzymes involved. CYP, cytochrome P450; FMO3, flavin-containing monooxygenase3 enzyme; N-desmethylclozapine, norclozapine; SULT’s, sulfotransferases; UGT’s, UDP-glucuronosyltransferases.

Clozapine is metabolized by *N*-demethylation and *N*-oxidation, yielding norclozapine and the inactive metabolite clozapine-*N*-oxide. The main enzymes involved are CYP1A2 and CYP3A4, as shown in Figure 2. Other Cytochrome P450 enzymes also contribute to *N*-demethylation^7,8^and *N*-oxidation.^8^

Norclozapine is further metabolized by conjugation with sulfate and glucuronic acid and eliminated in urine.^9^ It also undergoes tubular secretion^10^ by an unknown renal transporter.

Carbamazepine, in addition to clozapine therapy, is not recommended in some countries due to its additive risk of agranulocytosis. In the Dutch guideline for the use of clozapine (Dutch Clozapine Collaboration Group^11^), carbamazepine is mentioned as *relatively* contraindicated. Our patient did not improve on other mood stabilizers, memantine and numerous ECT sessions. Therefore, carbamazepine was started with frequent haematologic monitoring.

Because carbamazepine is a potent inducer of CYP1A2 and CYP3A4, the dose of clozapine has to be increased by a factor of 1.5–2.^12,13^ In this patient, this would have been a dose above 900 mg, which is the licenced maximum dose. Instead of increasing clozapine dose, fluvoxamine (a strong CYP1A2 inhibitor) was added to prevent clozapine levels to fall. Drug concentration levels were measured weekly, as shown in Figure 1.

Fluvoxamine strongly inhibited CYP1A2, increasing clozapine/norclozapine ratio (Figure 1, arrow 1). In this case clozapine/norclozapine ratio decreased and even inverted after addition of carbamazepine and increasing fluvoxamine dose to 50 mg. Norclozapine levels increased from 200 to 520 µg/l in 2 weeks.

### Several factors may have contributed to increasing norclozapine levels in this patient

When clozapine’s main metabolic pathway (CYP1A2) is inhibited by fluvoxamine, other metabolic pathways like CYP3A4 and CYP2C19 become more important. Carbamazepine is a strong inducer of several CYP450 enzymes, mainly CYP3A4, but also CYP1A2 and CYP2C19. CYP3A4 is involved in the formation of clozapine-*N*-oxide, as shown in Figure 2, but also of norclozapine,^7^ CYP2C19 is involved in the formation of norclozapine. Thus, more norclozapine and clozapine-*N*-oxide are formed when carbamazepine is added to clozapine treatment. Clozapine-*N*-oxide is not measured in routine daily practice.

Valproic acid is known to reduce norclozapine levels in clozapine-treated patients by presystemic induction of UGT enzymes or efflux transporters.^14^ Therefore, when valproic acid is discontinued, norclozapine levels can increase. After discontinuing an inducer, it can take several weeks till concentrations return to baseline, which would explain why norclozapine levels did not increase immediately after discontinuing valproic acid in this patient. The clozapine/norclozapine ratio increased from 1.99 to 3.15 after switching from lithium to valproic acid; this was mainly due to decreased norclozapine levels. This corroborates the suggestion that stopping the valproic acid has contributed to increasing norclozapine levels and inversion of the clozapine/norclozapine ratio.

The pharmacogenetic profile showed that the patient is an intermediate metabolizer for CYP2D6. The role of CYP2D6 in clozapine metabolism is not exactly known but it appears modest.^7,8^ Fluvoxamine is a substrate for CYP2D6. A small dose of fluvoxamine already can have a big impact on CYP1A2 metabolism in CYP2D6 intermediate metabolizers. In this case, the inducing effect by carbamazepine was stronger than the inhibition by fluvoxamine, which is in contrast with interaction data^12^ and a previously published case report about this complex interaction between clozapine, carbamazepine and fluvoxamine.^15^

### Several mechanisms may have contributed to the occurrence of paralytic ileus in this patient

First, antagonism of muscarinic M3 receptors in the smooth muscle of the colon may contribute to a reduction in colonic transit. Clozapine – a full antagonist of the M3 receptor – can cause gastrointestinal hypomotility.^16^ Although clozapine plasma concentrations may be good predictors of serum antimuscarinic activity,^17^ the association between clozapine plasma concentrations and constipation is unclear. A systematic review and meta-analysis of the association between clozapine and norclozapine serum levels and peripheral adverse drug reactions included seven studies regarding constipation.^18^ Six of the seven studies found no significant correlation between plasma clozapine levels and constipation. Only one study found that higher clozapine levels correlated with longer colonic transit times.^16^ So, antagonism of muscarinic receptors by higher clozapine levels might not be the key mechanism at stake in this patient.

A second proposed mechanism is antagonism of muscarinic M3 receptors by the partial M3 agonist norclozapine. Partial agonists act either as antagonists or as agonists, depending on the circumstances. They are also known as stabilizers.^19^ Norclozapine is a partial agonist of the M3 receptor, acting as either a full antagonist or a partial agonist, depending on cholinergic conditions. When cholinergic activity is low (when M3 receptors are blocked by clozapine or other anticholinergic drugs), norclozapine acts as partial agonist. But when cholinergic activity is high, a partial agonist acts as an antagonist.

This partial M3 agonism of norclozapine might explain an apparent paradox seen in the phenomenon of hypersalivation. Hypersalivation is mediated by stimulating muscarinic receptors. But clozapine, a full *antagonist* of the M3 receptor, is known to cause hypersalivation. Elevated clozapine plasma concentrations were observed in patients with hypersalivation.^20^ Our hypothesis is that norclozapine’s M3 agonism, occurring when clozapine levels are high, is the explanation for this intriguing paradox.

We hypothesize that this characteristic of norclozapine is also at stake in the occurrence of constipation. M3 *antagonism* can be a cause of constipation. One study reported a correlation between norclozapine levels and constipation.^21^ In our patient, constipation was getting worse while clozapine plasma levels decreased and norclozapine levels increased. Decreasing clozapine levels leads to increasing cholinergic activity, making norclozapine act as an antagonist of M3, causing constipation.

Our hypothesis is supported by the finding that hypersalivation was gone after stopping glycopyrronium tablets – norclozapine no longer acts as an agonist and no longer causes hypersalivation. So, when both clozapine and norclozapine act as antagonists at the M3 receptor, more constipation is expected.

Prevalence of constipation is approximately three times higher in clozapine users than in users of other antipsychotics. This also includes other anticholinergic antipsychotics such as olanzapine.^2^ Therefore, it is very likely that antagonism of muscarinic receptors is not the only mechanism involved. A third proposed mechanism and possible explanation for the higher prevalence of constipation during clozapine treatment is norclozapine’s activity on opioid receptors.^21^

Norclozapine acts as a selective and efficacious delta-opioid receptor agonist. The parent drug, clozapine, also can activate delta-opioid receptors, but with a 10-fold lower affinity and a low efficacy compared to norclozapine.^22^ In Chinese hamster ovary cells expressing the delta-opioid receptor, norclozapine was a full agonist as measured by GTPγS binding assay (pEC50 = 7.24), clozapine exhibited a potency approximately 10-fold lower (pEC50 = 5.9). In the Psychoactive Drug Screening Program (PDSP) K_i_-database a K_i_ of 127.6 nM is reported for norclozapine (binding affinity at cloned human receptors).^23^ This corresponds with a norclozapine concentration of 40 µg/l, which is below the concentrations measured in patients on clozapine therapy. For clozapine, the PDSP database only shows a K_i_ in mouse cells (1,000 nM). Although it is difficult to compare K_i_’s in different species, this indicates a much lower binding affinity. Opioids are known to cause constipation. Another study showed that delta-opioid receptor antagonist naltrindole reduces oxycodone addiction and constipation in mice.^24^ In our patient, norclozapine plasma levels increased by a factor of 2.5 before hospital admission. It is very likely that norclozapine’s pharmacological activity has contributed to the paralytic ileus. Norclozapine’s action on the delta-receptor needs further investigation because it can offer therapeutic options to treat clozapine-induced constipation with delta-antagonist in the future.

Finally, clozapine-*N*-oxide and carbamazepine itself may have contributed to the occurrence of constipation. Carbamazepine has mild anticholinergic properties. However, the onset of constipation occurred 2 months after the introduction of carbamazepine, which makes a direct causative relationship less plausible. We performed a Naranjo scale analysis^25^ to assess the causal relationship between the adverse event and either clozapine or carbamazepine. The score was 7 (probable) for clozapine and 2 (possible) for carbamazepine (Supplemental Table 1). Clozapine has the highest score, although in this case the adverse drug reaction was not directly related to clozapine itself, but rather stemmed from the altered metabolism resulting from a pharmacokinetic interaction.

The absence of routine measurement of clozapine-*N*-oxide concentration poses a limitation in our case report. This omission deprives us of essential information concerning possible changes in this metabolite’s levels and its potential contribution to the onset of constipation. However, the main strength of our case report is repeated measurement of clozapine and norclozapine levels, enabling us to track changes following each adjustment in medication.

The clozapine/norclozapine ratio has been studied mainly in relation to cognition and metabolic side effects. So far, a positive association between the clozapine/norclozapine ratio and better cardiometabolic outcomes is suggested.^26^ On the other hand, a higher ratio is correlated with lower cognitive performance in most studies.^27,26^ There are no studies about the association between the clozapine/norclozapine ratio and constipation.

## Conclusion

Our patient developed paralytic ileus after substituting valproic acid with carbamazepine (+fluvoxamine) during clozapine therapy. Clozapine levels decreased, and norclozapine levels increased, resulting in an inverted clozapine/norclozapine ratio.

We discussed antagonism of M3 receptors by clozapine and norclozapine and agonism of delta-opioid receptors by norclozapine as possible mechanisms that have contributed to the occurrence of the paralytic ileus in our patient. We propose that both the clozapine/norclozapine ratio and the absolute norclozapine levels can be considered risk factors for constipation and paralytic ileus.

There is growing evidence that the clozapine/norclozapine ratio is important in cognition and metabolic side effects of clozapine, but this is the first report on the potential impact of the clozapine/norclozapine ratio on constipation and paralytic ileus.

## Supplemental Material

sj-pdf-1-tpp-10.1177_20451253241255487 – Supplemental material for Paralytic ileus in a patient on clozapine therapy showing an inverted clozapine/norclozapine ratio after switching valproic acid to carbamazepine: a case report Supplemental material, sj-pdf-1-tpp-10.1177_20451253241255487 for Paralytic ileus in a patient on clozapine therapy showing an inverted clozapine/norclozapine ratio after switching valproic acid to carbamazepine: a case report by Geke van Weringh, Leonieke van Koolwijk, Lieuwe de Haan, Daan J. Touw and Mariken B. de Koning in Therapeutic Advances in Psychopharmacology

sj-pdf-2-tpp-10.1177_20451253241255487 – Supplemental material for Paralytic ileus in a patient on clozapine therapy showing an inverted clozapine/norclozapine ratio after switching valproic acid to carbamazepine: a case report Supplemental material, sj-pdf-2-tpp-10.1177_20451253241255487 for Paralytic ileus in a patient on clozapine therapy showing an inverted clozapine/norclozapine ratio after switching valproic acid to carbamazepine: a case report by Geke van Weringh, Leonieke van Koolwijk, Lieuwe de Haan, Daan J. Touw and Mariken B. de Koning in Therapeutic Advances in Psychopharmacology

## Declarations

**Ethics approval and consent to participate:** Ethics approval is not required for case reports at Arkin Institute for Mental Healthcare, where the patient stayed.

**Consent for publication:** The patient’s curator provided written informed consent for the publication of this case report.

**Availability of data and materials:** Not applicable.

## References

[1] Siskind D McCartney L Goldschlager R , et al. Clozapine v. first- and second-generation antipsychotics in treatment-refractory schizophrenia: systematic review and meta-analysis. Br J Psychiatry 2016; 209: 385–392. 27388573 10.1192/bjp.bp.115.177261

[2] Shirazi A Stubbs B Gomez L , et al. Prevalence and predictors of clozapine-associated constipation: a systematic review and meta-analysis. Int J Mol Sci 2016; 17: 863. 27271593 10.3390/ijms17060863 PMC4926397

[3] Xu Y Amdanee N Zhang X. Antipsychotic-induced constipation: a review of the pathogenesis, clinical diagnosis, and treatment. CNS Drugs 2021; 35: 1265–1274. 34427901 10.1007/s40263-021-00859-0

[4] De Leon J Sanz EJ De las Cuevas C . Data from the World Health Organization’s pharmacovigilance database supports the prominent role of pneumonia in mortality associated with clozapine adverse drug reactions. Schizophr Bull 2020; 46: 1–3. 31901099 10.1093/schbul/sbz093 PMC6942151

[5] Cohen D. Clozapine and gastrointestinal hypomotility. CNS Drugs 2017; 31: 1083–1091. 29230675 10.1007/s40263-017-0481-5

[6] Gagnier JJ Kienle G Altman DG , et al. The CARE guidelines: consensus-based clinical case reporting guideline development. BMJ Case Rep 2013; 2013: bcr-2013-201554. 10.1186/1752-1947-7-223 PMC3844611 24228906

[7] Olesen OV Linnet K. Contributions of five human cytochrome P450 isoforms to the N-demethylation of clozapine in vitro at low and high concentrations. J Clin Pharmacol 2001; 41: 823–832. 11504269 10.1177/00912700122010717

[8] PHARMGKB, https://www.pharmgkb.org/pathway/PA166163661 (accessed January 2024).

[9] Schaber G Wiatr G Wachsmuth H , et al. Isolation and identification of clozapine metabolites in patient urine. Drug Metab Dispos 2001; 29: 923–931. 11353764

[10] Schaber G Stevens I Gaertner HJ , et al. Pharmacokinetics of clozapine and its metabolites in psychiatric patients: plasma protein binding and renal clearance. Br J Clin Pharmacol 1998; 46: 453–459. 9833598 10.1046/j.1365-2125.1998.00822.x PMC1873700

[11] Guideline for the use of clozapine by the Dutch Clozapine Collaboration Group, https://www.clozapinepluswerkgroep.nl (2013, accessed January 2024).

[12] Spina E Hiemke C de Leon J. Assessing drug-drug interactions through therapeutic drug monitoring when administering oral second-generation antipsychotics. Expert Opin Drug Metab Toxicol 2016; 12: 407–422. 26878495 10.1517/17425255.2016.1154043

[13] Jerling M Lindström L Bondesson U , et al. Fluvoxamine inhibition and carbamazepine induction of the metabolism of clozapine: evidence from a therapeutic drug monitoring service. Therapeutic Drug Monit 1994; 16: 368–374. 10.1097/00007691-199408000-00006 7974626

[14] Smith RL Kyllesø L Haslemo T , et al. Reduction in N-Desmethylclozapine level is determined by daily dose but not serum concentration of valproic acid—indications of a presystemic interaction mechanism. Therapeutic Drug Monit 2019; 41: 503–508. 10.1097/FTD.0000000000000619 31259880

[15] Chen W-Y Shen Y-C. Complex drug interaction of carbamazepine, fluvoxamine and clozapine in a patient with bipolar depression. Archives Clin Psychiatry (São Paulo) 2019; 46: 171–171.

[16] Every-Palmer S Nowitz M Stanley J , et al. Clozapine-treated patients have marked gastrointestinal hypomotility, the probable basis of life-threatening gastrointestinal complications: a cross sectional study. EBioMedicine 2016; 5: 125–134. 27077119 10.1016/j.ebiom.2016.02.020 PMC4816835

[17] de Leon J Odom-White A Josiassen RC , et al. Serum antimuscarinic activity during clozapine treatment. J Clin Psychopharmacol 2003; 23: 336–341. 12920408 10.1097/01.jcp.0000085405.08426.73

[18] Tan MSA Honarparvar F Falconer JR , et al. A systematic review and meta-analysis of the association between clozapine and norclozapine serum levels and peripheral adverse drug reactions. Psychopharmacology (Berl) 2021; 238: 615–637. 33410989 10.1007/s00213-020-05746-y

[19] Stahl SM. Stahl’s essential psychopharmacology: neuroscientific basis and practical applications. Cambridge: Cambridge University Press, 2021.

[20] Schoretsanitis G Kuzin M Kane JM , et al. Elevated clozapine concentrations in clozapine-treated patients with hypersalivation. Clin Pharmacokinet 2021; 60: 329–335. 33000411 10.1007/s40262-020-00944-5

[21] Bailey L Varma S Ahmad N , et al. Factors predicting use of laxatives in outpatients stabilized on clozapine. Ther Adv Psychopharmacol 2015; 5: 256–262. 26557981 10.1177/2045125315591917 PMC4622118

[22] Onali P Olianas MC. N-Desmethylclozapine, a major clozapine metabolite, acts as a selective and efficacious delta-opioid agonist at recombinant and native receptors. Neuropsychopharmacology 2007; 32: 773–785. 16841075 10.1038/sj.npp.1301152

[23] PDSP K_i_-database, https://pdsp.unc.edu/pdspweb/ (accessed January 2024).

[24] Yang P-P Yeh T-K Loh HH , et al. Delta-opioid receptor antagonist naltrindole reduces oxycodone addiction and constipation in mice. Eur J Pharmacol 2019; 852: 265–273. 30959048 10.1016/j.ejphar.2019.04.009

[25] Naranjo CA Busto U Sellers EM , et al. A method for estimating the probability of adverse drug reactions. Clin Pharmacol Ther 1981; 30: 239–245. 7249508 10.1038/clpt.1981.154

[26] Costa-Dookhan KA Agarwal SM Chintoh A , et al. The clozapine to norclozapine ratio: a narrative review of the clinical utility to minimize metabolic risk and enhance clozapine efficacy. Expert Opin Drug Saf 2020; 19: 43–57. 31770500 10.1080/14740338.2020.1698545

[27] Schoretsanitis G Kane JM Ruan C-J , et al. A comprehensive review of the clinical utility of and a combined analysis of the clozapine/norclozapine ratio in therapeutic drug monitoring for adult patients. Expert Rev Clin Pharmacol 2019; 12: 603–621. 31075044 10.1080/17512433.2019.1617695

