# Supplementary material for: Paralytic ileus in a patient on clozapine therapy showing an inverted clozapine/norclozapine ratio after switching valproic acid to carbamazepine: a case report
Source: Ther Adv Psychopharmacol. 2024 May 31;14:20451253241255487. doi: 10.1177/20451253241255487 (PMC11143807; doi:10.1177/20451253241255487)
Supplement: sj-pdf-1-tpp-10.1177_20451253241255487 – Supplemental material for Paralytic ileus in a patient on clozapine therapy showing an inverted clozapine/norclozapine ratio after switching valproic acid to carbamazepine: a case report [file sj-pdf-1-tpp-10.1177_20451253241255487.pdf]

CARE case report guidelines

CARE Checklist of information to include when writing a case report

(cc) BY-NC-ND

Topic	Item	Checklist item description	Reported on Line
Title	1	The diagnosis or intervention of primary focus followed by the words "case report"	p2, line 7
Key Words	2	2 to 5 key words that identify diagnoses or interventions in this case report, including "case report"	p3, line 12
Abstract (no references)	3a	Introduction: What is unique about this case and what does it add to the scientific literature?	p2, line 39
	3b	Main symptoms and/or important clinical findings	p2
	3c	The main diagnoses, therapeutic interventions, and outcomes	p2, lines 40-59
	3d	Conclusion—What is the main "take-away" lesson(s) from this case?	p3, line 6
Introduction	4	One or two paragraphs summarizing why this case is unique (**may include references**)	p3, lines 17-34
Patient Information	5a	De-identified patient specific information.	p3, p4
	5b	Primary concerns and symptoms of the patient.	p3, lines 43-53
	5c	Medical, family, and psycho-social history including relevant genetic information	p3,line 45 - p4,line27
	5d	Relevant past interventions with outcomes	p3,line55 - p4,line 23
Clinical Findings	6	Describe significant physical examination (PE) and important clinical findings.	p4, lines 16-27
Timeline	7	Historical and current information from this episode of care organized as a timeline	text p3, p4 + figure 1
Diagnostic Assessment	8a	Diagnostic testing (such as PE, laboratory testing, imaging, surveys).	p4,lines 11-16 and 24-26
	8b	Diagnostic challenges (such as access to testing, financial, or cultural)	N/A
	8c	Diagnosis (including other diagnoses considered)	p3,lines 43-53 and p4,16-22
	8d	Prognosis (such as staging in oncology) where applicable	N/A
Therapeutic Intervention	9a	Types of therapeutic intervention (such as pharmacologic, surgical, preventive, self-care)	p4, lines 28-29
	9b	Administration of therapeutic intervention (such as dosage, strength, duration)	p4, lines 29-40 and 57-59
	9c	Changes in therapeutic intervention (with rationale)	N/A
Follow-up and Outcomes	10a	Clinician and patient-assessed outcomes (if available)	p4,41-46+p4,line 54-p5,line10
	10b	Important follow-up diagnostic and other test results	p4,lines 47-53+pg 5, lines 8-9
	10c	Intervention adherence and tolerability (How was this assessed?)	p4, lines 38-39
	10d	Adverse and unanticipated events	p4, lines 41-46
Discussion	11a	A scientific discussion of the strengths AND limitations associated with this case report	p7, line 46-51
	11b	Discussion of the relevant medical literature **with references**.	p5, p6, p7
	11c	The scientific rationale for any conclusions (including assessment of possible causes)	p7, lines15-29 + lines 39-44
	11d	The primary "take-away" lessons of this case report (without references) in a one paragraph conclusion	page 8
Patient Perspective	12	The patient should share their perspective in one to two paragraphs on the treatment(s) they received	N/A
Informed Consent	13	Did the ~~patient~~ give informed consent? Please provide if requested the curator of the patient	Yes ☒ No ☐
