# Supplementary material for: Paralytic ileus in a patient on clozapine therapy showing an inverted clozapine/norclozapine ratio after switching valproic acid to carbamazepine: a case report
Source: Ther Adv Psychopharmacol. 2024 May 31;14:20451253241255487. doi: 10.1177/20451253241255487 (PMC11143807; doi:10.1177/20451253241255487)
Supplement: sj-pdf-2-tpp-10.1177_20451253241255487 – Supplemental material for Paralytic ileus in a patient on clozapine therapy showing an inverted clozapine/norclozapine ratio after switching valproic acid to carbamazepine: a case report [file sj-pdf-2-tpp-10.1177_20451253241255487.pdf]

Supplementary table 1. Naranjo Adverse Drug Reaction Probability Scale for clozapine and carbamazepine

	yes	no	do not know	**clozapine**		**carbamazepine**	
Are there previous conclusive reports on this reaction?	1	0	0	yes	1	no	0
Did the adverse event appear after the suspected drug was administered?	2	-1	0	yes	2	yes	2
Did the adverse event improve when the drug was discontinued or a specific antagonist was administered?	1	0	0	N/A	0	N/A	0
Did the adverse event reappear when the drug was readministered?	2	-1	0	N/A	0	N/A	0
Are there alternative causes that could on their own have caused this reaction?	-1	2	0	no	2	yes	-1
Did the reaction reappear when a placebo was given?	-1	1	0	N/A	0	N/A	0
Was the drug detected in blood or other fluids in concentrations known to be toxic?	1	0	0	no	0	no	0
Was the reaction more severe when the dose was increased or less severe when the dose was decreased?	1	0	0	no	0	no	0
Did the patient have a similar reaction to the same or similar drugs in any previous exposure?	1	0	0	yes	1	no	0
Was the adverse event confirmed by any objective evidence?	1	0	0	yes	1	yes	1
**Total Score**					**7**		**2**
